# Stent thrombosis caused by metal allergy complicated by protein S deficiency and heparin-induced thrombocytopenia: a case report and review of the literature

**DOI:** 10.1186/s12959-015-0055-z

**Published:** 2015-07-23

**Authors:** Takao Konishi, Tadashi Yamamoto, Naohiro Funayama, Beni Yamaguchi, Seiichiro Sakurai, Hiroshi Nishihara, Koko Yamazaki, Yusuke Kashiwagi, Yasuki Sasa, Mitsuru Gima, Hideichi Tanaka, Daisuke Hotta, Kenjiro Kikuchi

**Affiliations:** Department of Cardiology, Hokkaido Cardiovascular Hospital, 1-30, West 13, South 27, Chuou-ku, Sapporo, 064-8622 Japan; Department of Translational Pathology, Hokkaido University School of Medicine, Sapporo, Japan

**Keywords:** Stent thrombosis, Metal allergy, Heparin-induced thrombosis, Protein S deficiency

## Abstract

A 43-year-old woman recipient of a bare metal coronary stent during an acute anterior myocardial infarction was repeatedly hospitalized with recurrent stent thrombosis (ST) over the following 3 years. Emergent coronary angiography showed a thrombus in the in-stent segment of the proximal left anterior descending artery. We repeatedly aspirated the thrombus, which immediately reformed multiple times. The discontinuation of heparin and administration of thrombolytics and argatroban, followed by repeated balloon dilatations, ended the formation of new thrombi. The patient was found to be allergic to nickel, protein S deficient and carrier of heparin-induced thrombocytopenia antibody. We discuss this case in the context of a) literature pertaining to acute coronary syndromes in the young, and b) the detailed investigations needed to identify thrombotic risk factors. Steroids may be effective to prevent recurrent ST caused by stent allergy.

## Background

Stent thrombosis (ST) is a rare though critical complication of PCI, which may occur despite recent progress in antiplatelet therapy and procedural techniques. The incidence of ST ranges between 0.6 and 4.4 % [1–5]. However, the clinical consequences of ST include myocardial infarction in 70 % to 80 % or death in 30 % of cases [6]. Therefore, ST remains an important clinical challenge in the modern era of stent deployment. There are many patient-related potential factors of ST such as smoking, diabetes mellitus, chronic kidney disease and premature discontinuation or cessation of dual antiplatelet therapy [7]. So far, however, no reports exist mentioning ST caused by metal allergy complicated by protein S deficiency and heparin-induced thrombocytopenia.

## Case presentation

A 43-year-old woman was admitted to the coronary care unit, complaining of prolonged chest pain that developed while playing a mobile game at home. Acute coronary syndrome (ACS) was diagnosed from the electrocardiogram, which showed a 1-mm ST segment elevation in the precordial leads V1–V4 and reciprocal, 1-mm ST segment depression in leads I and aVL, with abnormal Q waves in V1–V3. Her coronary risk factors were dyslipidaemia and current smoking. She had undergone a) implantation of a 4.0 × 18.0 mm Driver™ (Medtronic, Inc., Minneapolis, MN) bare metal stent in the proximal left anterior descending (pLAD) artery 3 years earlier for management of a first ACS with myocardial infarction, and b) percutaneous coronary intervention (PCI) and thrombus aspiration 12 months later for treatment of a 2nd ACS due to in-stent thrombosis. After having being treated with warfarin for 2 years to prevent ST, the international normalized ratio had fallen between 1.1 and 1.3 (below the optimal therapeutic range), because of anaemia due to abundant menstrual haemorrhages, and she was re-hospitalized for treatment of her 3rd ACS.

**Fig. 1:**
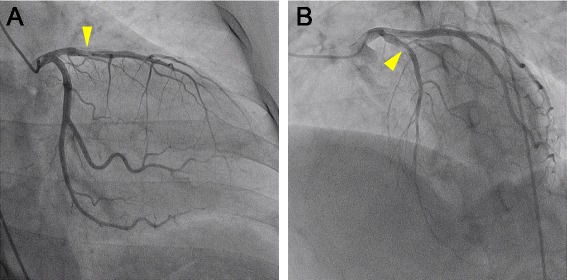
Coronary angiography. **a**. Right anterior oblique and caudal view. **b**. Left anterior oblique and cranial view. A stent thrombosis is visible (arrowheads) in the in-stent segment of the proximal left anterior descending artery

**Fig. 2:**
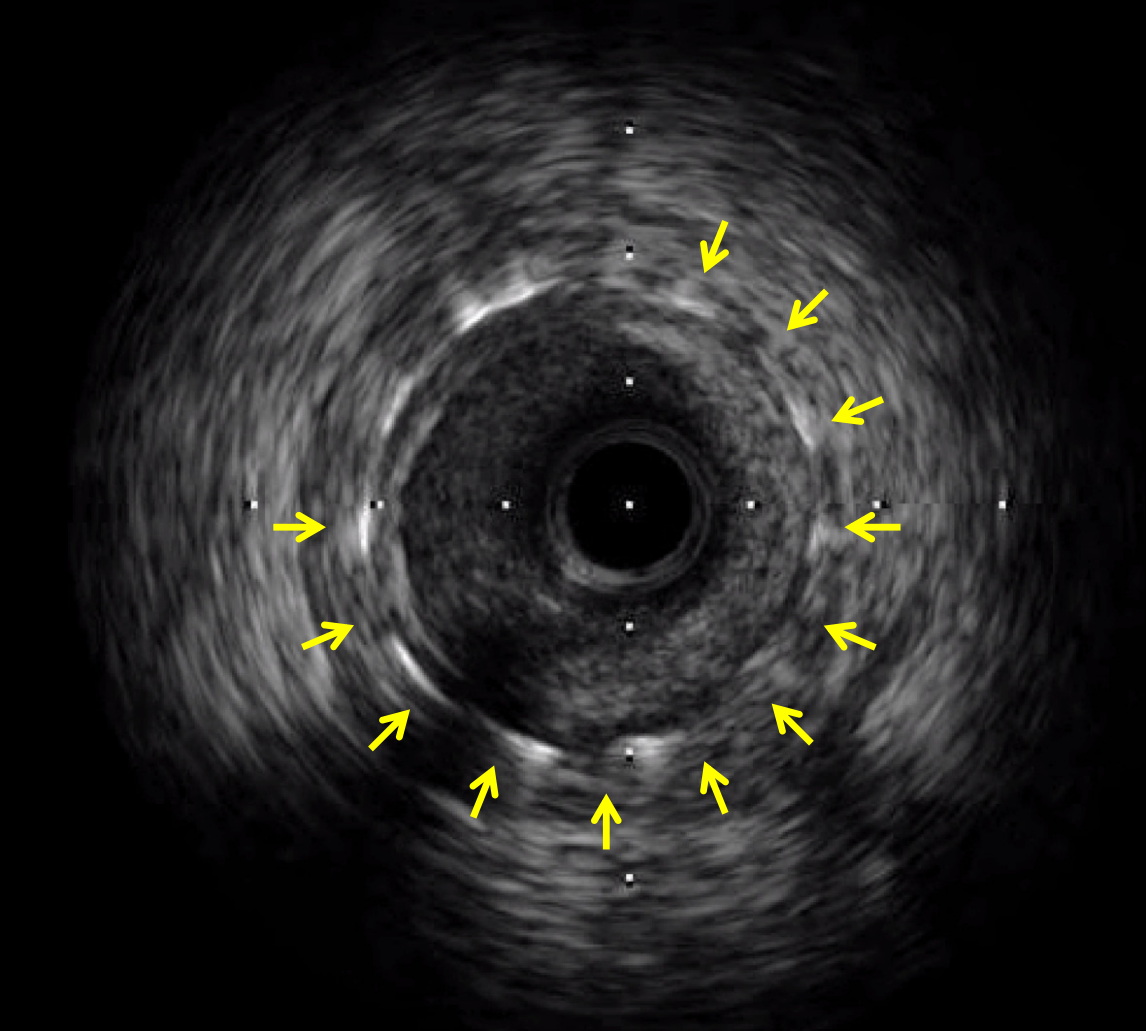
Intravascular ultrasound. Fibrous thrombi are visible in the in-stent segment of the pLAD artery

**Fig. 3:**
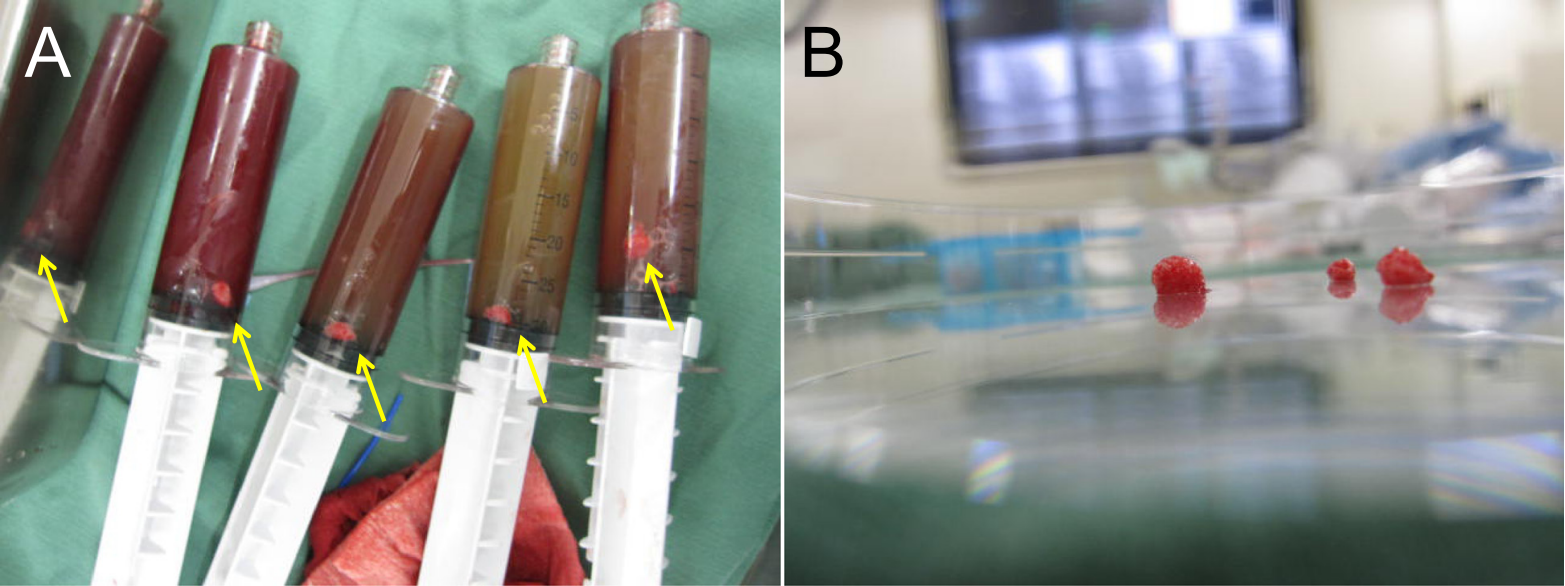
Aspirated thrombi. **a**. Several large thrombi (arrow) were aspirated during PCI and preserved in physiologic saline inside the aspiration device. **b**. Extracted thrombi on a Petri dish

On physical examination, her pulse was 80 bpm, blood pressure 128/83 mmHg, and a 3rd heart sound was audible on auscultation. The laboratory tests revealed a 21.0 × 10^3^/μl white blood cell count with 96.5 % neutrophils, a platelet count of 42.2 × 10^4^/μl, a 7.3 g/dl haemoglobin concentration, 63.0 and 5.3 IU/l, CK and CK-MB concentrations, respectively, negative H-FABP, and 0.8 μg/ml d-dimer and 0.02 mg/dl C-reactive protein serum concentrations. She immediately received 5,000 units of unfractionated heparin in a single bolus, followed by another 5,000 units bolus in the catheterization laboratory, before undergoing emergent coronary angiography, which revealed the presence of stent thrombosis (ST) and TIMI 2 distal flow in the pLAD artery (Fig. 1). An intravascular ultrasound study showed an in-stent fibrous thrombus in the pLAD artery (Fig. 2). After each aspiration of the thrombus with a special catheter, a large, red thrombus reappeared, requiring 10 consecutive aspirations over 45 min (Fig. 3). Suspecting heparin-induced thrombocytopenia (HIT), we discontinued the administration of heparin and substituted argatroban. Balloon angioplasty was performed, without further recurrence of thrombus, and the patient, finally chest pain free, stabilized clinically and was discharged from the hospital on the 27th day on a regimen of dabigatran instead of warfarin.

During her hospitalization, the patient underwent investigations of the cause of ST, including a patch test, blood coagulation test and HIT antibody. The skin patch test showed a positive response to nickel, a metal included in the composition of the Driver stent. Protein C, antithrombin III, antinuclear antibody, anticardiolipin 2-glycoprotein I complex antibody and lupus anticoagulant were within normal limits, though the concentration of protein S (PS) was 35 % (normal = 60–150 %), total PS antigen was 56 % (normal = 65–135 %) and free PS antigen was 50 % (normal = 60–150 %). PS deficiency is classified as type I (decreased concentration of total and free PS antigen), type II (decreased activity of activated protein C-cofactor and normal total and free PS antigen concentration), and type III (decreased concentration of free PS antigen only) [8]. This patient presented with type I PS deficiency. The HIT test showed a weakly positive reaction: IgG・IgM・IgA 1.3 U/ml (normal ≤1.0 U/ml). Intravascular ultrasound study during PCI showed no incomplete stent apposition or stent fracture. The patient had been compliant with antiplatelet therapy before her admission to the hospital, including aspirin 100 mg/day and clopidogrel 75 mg/day, and was not using oral contraception.

The patient was discharged from the hospital on a regimen of dabigatran, 300 mg/day, later replaced by rivaroxaban, 15 mg/day, instead of warfarin. However, she suffered a 4th ST in the pLAD artery 4 months later and, despite the prescription of the anti-allergic agent, fexofenadine, 120 mg/day, she experienced a 5th ST 1 month later. Prednisolone, gradually tapered from 25 to 5 mg/day, was added and the patient remained free from ST over a >18-month follow-up. This clinical course suggested that her recurrent thrombotic events were caused mainly by a stent allergy, and that PS deficiency and HIT are associated with ST.

## Discussion

The pathophysiology of ST includes lesion-, stent-, procedure-, and patient-related factors. Early ST are often associated with lesion—or procedural factors, such as insufficient expansion of the stent, edge dissection, compromised flow and location of the stent at a bifurcation [9]. On the other hand, late and very late ST are associated with a variety of causes, such as local hypersensitivity reaction, poor endothelialization, inflammation, delayed healing and neoatherosclerosis [10, 11]. To the best of our knowledge, this is the first report of ST caused by metal allergy complicated by PS deficiency and HIT in a young woman.

### Stent allergy

ST or in-stent restenosis (ISR) due to stent allergy have been reported [12–15]. Aliagaoglu et al. reported nickel allergies confirmed by patch tests in 7 of 31 patients (23 %) who developed ISR, while none of 30 patients free from ISR had an allergy to nickel (p < 0.01) [12]. Koster et al. reported that 10 of 89 patients (11 %) who developed restenosis after PCI had positive reactions to nickel or molybdenum, which are standard components of metal stents [14]. Furthermore, the prevalence of nickel allergy in the general population is as high as 17 % in women and 3 % in men [16]. These observations suggest that stent allergy is an important risk factor of ST.

The mechanism of ST caused by metal allergy is a local endothelial inflammation and excessive immune response. After the implantation of a stent that contains nickel, the metal is steadily released into the systemic circulation, promoting the expression of intercellular adhesion molecule-1 on endothelial cells, which plays an important role in the recruitment of inflammatory cells from the bloodstream [17, 18]. In addition, local exposure to a stent containing nickel causes a type IV hypersensitivity mediated by allergen-specific T lymphocytes, which can trigger excessive immunologic reactions [19, 20]. Once nickel sensitization has occurred, it may persist for many years [21]. Since the bare metal stent implanted in this patient contained cobalt, nickel, chromium and molybdenum, a risk of ST was present since the stent implantation 3 years earlier. Therefore, patients who develop repetitive ST after implantation should undergo patch testing in search of metal allergy.

**Fig. 4:**
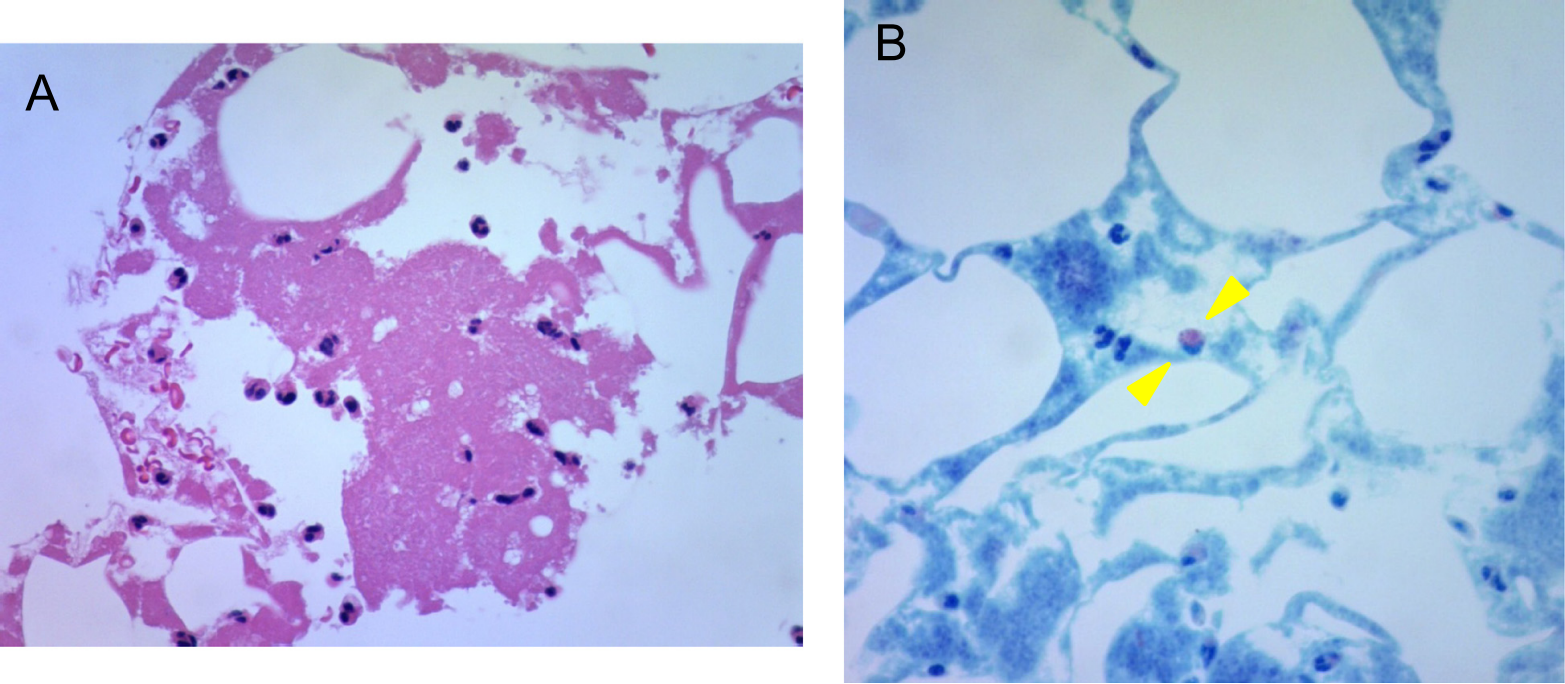
Histological examination of aspirated thrombi. **a**. High-power (original magnification 400×) microphotograph showing the infiltration of inflammatory cells in aspirated fibrin-rich thrombus as observed after haematoxylin and eosin staining. **b**. High-power (original magnification 400×) microphotograph showing the eosinophilic infiltration as observed after Giemsa staining (between arrowheads)

A significant increase in white blood cells, including eosinophils, does not occur systematically after implantation of coronary stents in metal allergic patients presenting with ISR [22]. Because of a paucity of Langerhans cells compared to the skin, the placement of stents in the coronary vessels triggers a weak immunological response [21]. Therefore, it is likely that, as illustrated in this case, an allergy to a metallic stent triggers an increase in eosinophil count consistent with a local instead of a systemic allergic reaction. In a *post mortem* study, eosinophilic infiltrations were observed at the site of the stent in patients with ST associated with stent allergies [23]. In our case, the histology showed eosinophilic infiltrates in part of the aspirated thrombus (Fig. 4), suggesting that a local stent allergic reaction was associated with ST.

Since inflammation is one of the main causes of ST or ISR [24, 25], the systemic administration of anti-inflammatory or immunosuppressive therapy might be appropriate when a metal allergy is confirmed or strongly suspected. The implantation of a stent causes a prolonged recruitment of inflammatory cells [26]. In recent studies, the administration of oral steroids after PCI suppressed the vascular inflammation and lowered the rates of ST, ISR, or both [27–33]. For example, in the CEREA-DES trial, the adverse event-free survival of 125 recipients of bare metal stent alone was 75 %, versus 84 % in 125 prednisone-treated patients, a significant difference [32]. Chronic inflammation and endothelial dysfunction induce neoatherosclerosis on the long term inside both bare metal stent and drug eluting stent (DES), and the disruption of neoatherosclerotic plaques plays an important role in the occurrence of late, and especially, very late ST [11, 34–36]. Considering that, in our patient, ST developed 5 times since the stent implantation, 3 years earlier, we hypothesize that a local inflammation caused by stent allergy promoted a persistent thrombogenic propensity and multiple thrombotic events during that period.

While warfarin or new oral antithrombotic agents must be administered in patients who develop ST after PCI [37–39], low-dose steroids in addition to dual antiplatelet therapy might prove effective, especially for patients who develop ST due to stent allergy, provided they do not suffer from diabetes or other contraindications to steroids. Unlike pharmacologic doses of glucocorticoids, which seem to increase the risk of adverse cardiovascular events [40], low doses of steroids might confer clinical benefits and lower the incidence of ST by their anti-inflammatory properties. The relative risk of adverse cardiovascular events in recipients of ≥7.5 mg of prednisolone equivalent on the long term was 2.56 [40]. In contrast, in another study, oral prednisone lowered significantly the cumulative incidence of major adverse cardiovascular events, including ST [32]. The prednisone regimen consisted of 1 mg/kg for the first 15 days after stent implantation, 0.5 mg/kg from day 16 to day 30, and 0.25 mg/kg from day 31 to day 40. In this patient, an initial dose of 25 mg/day of prednisolone was tapered by 5 mg every month to a maintenance dose of 5 mg/day. The dose of prednisolone remained unchanged and no further ST was observed.

Stent extraction followed by coronary artery bypass graft is an alternate means of management of ST due to stent allergy [41–43]. Likewise, recent studies have reported a low incidence of ST after implantation of bioresorbable vascular scaffolds, which may be particularly useful in patients presenting with stent allergy undergoing PCI [44, 45]. After the elution of the anti-proliferative drug and the resorption of the scaffold, the risk of ST caused by metal allergy is markedly decreased. A direct comparison of anticoagulation versus resorbable stent versus steroid therapy would be of great interest to determine which is the most effective prevention of ST associated with stent allergy.

### Heparin-induced thrombocytopenia

The incidence of heparin-induced thrombocytopenia (HIT) ranges between 0.3 and 5.0 % of patients exposed to unfractionated heparin [46–48]. Women are more likely than men to develop HIT with an approximate relative risk of 1.5 to 2.0 [49, 50]. HIT is closely associated with thrombotic events, with a 20–40 odds ratio for thrombosis, and a thrombotic risk ranging between 38 and 76 % [50] and a 10.2 % mortality rate [51]. Therefore, a prompt diagnosis of HIT is key when patients administered heparin present with thrombocytopenia or thrombosis.

**Fig. 5:**
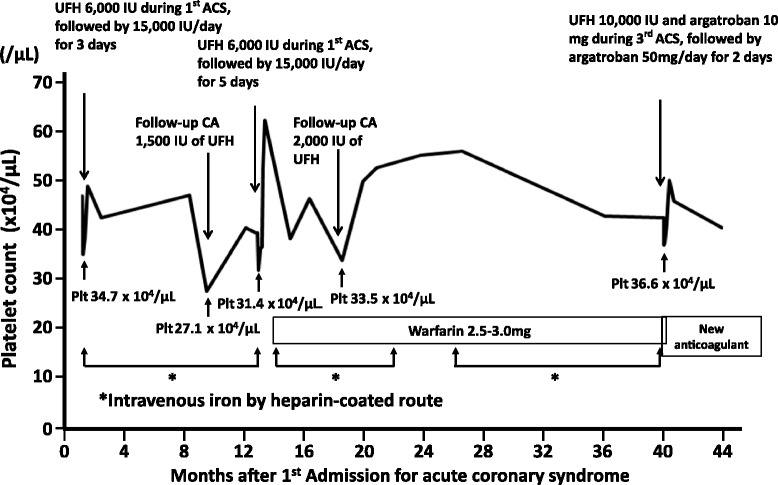
Evolution of platelet count. The platelet count decreased after each administration of heparin for emergent PCI or follow-up coronary angiography. However, the fall in platelet count was not recognized until the 3rd hospitalization for ACS as the baseline platelet count was high and the lowest count during each admission remained within normal limits. The patient received iron intravenously nearly every month for 3 years for profuse menstrual bleeding, which might have activated circulating HIT antibodies. This patient received 10,000 IU of total unfractionated heparin during PCI on the third ACS, higher than during previous angiographies or PCI (1,500–6,000 IU). ACS = acute coronary syndrome; CA = coronary angiography; UFH = unfractionated heparin; Plt = platelet

While the platelet count in this patient was within normal limit, the relative change in platelets instead of the absolute platelet count must be ascertained [52, 53]. The evolution of our patient’s platelet count is shown in Fig. 5. A “4T” scoring system, widely used for the clinical diagnosis of HIT, includes 1) the degree of Thrombocytopenia, 2) the Timing of the fall in platelet count after heparin exposure, 3) the presence of Thrombosis, and 4) the exclusion of oTher causes of thrombocytopenia [54]. Our patient’s 4T score was 5 out of 8, consistent with an intermediate probability of HIT. The positive predictive value of an intermediate 4T score in a population at relatively high risk of HIT is approximately 45 % [55]. The HIT Expert Probability score, based on the opinions of 26 expert clinicians [56], is a new pre-test probability model, which, using a cut-off score of 5, has been associated with a 100 % sensitivity, 60 % specificity and 55 % positive predictive value in the detection of HIT [56]. The likelihood of HIT in our patient seemed relatively high, as her calculated score was 9. Her clinical presentation was also consistent with a relationship between HIT and acute coronary thrombosis during PCI. The administration of argatroban after the discontinuation of heparin prevented the formation of new thrombi during PCI. Furthermore, the platelet count decreased consistently when she was hospitalized and was administered heparin during emergent PCI or follow-up coronary angiography (Fig. 5). However, because her baseline platelet count was high and the lowest counts remained within normal limits, the cause of transient fall in platelet count was not recognized and investigated until the third hospitalization for ACS.

In presence of circulating HIT antibodies from a recent heparin exposure, a rapid-onset type of HIT usually develops within minutes or hours after the beginning of heparin treatment. Once the antibodies are formed, they may persist for up to 100 days after the administration of heparin [57]. Since the last ACS occurred approximately 2 years earlier, this seemed to be an atypical case of rapid onset. However, the minuscule doses of heparin contained in the heparin-coated infusions or heparin flushes of intravenous iron preparation she had received nearly monthly for abundant menstrual bleeding (Fig. 5) might have unknowingly activated the HIT antibodies.

HIT antibodies may be activated more prominently by high than by low doses of intravenous heparin, although this has not been prospectively examined in a large study. In a study by Chong, the incidence of HIT ranged between 1 and 30 % among patients who received high doses of intravenous heparin, in contrast with <2 % in patients who received low doses of subcutaneous heparin [58]. Our patient received a bolus of 10,000 IU of unfractionated heparin during the PCI for the 3rd ACS, compared with 1,500–6,000 IU during the previous coronary angiograms or PCI (Fig. 5). This different dose of heparin might explain why a ST occurred during that PCI, and did not occur during the previous angiograms or PCI.

Since they interact with neither platelet factor 4 (PF4) nor anti-PF4/heparin antibodies, the new oral antithrombotics may be a valuable therapeutic alternative for patients who present with a history of HIT [59–61]. In our patient, warfarin was replaced by dabigatran at the time of hospital discharge and, later, dabigatran was replaced by rivaroxaban to promote compliance with treatment.

### Protein S deficiency

The estimated prevalence of PS deficiency in the general population is 1–2 % [62–64]. PS is a plasma protein that serves as a cofactor for the anticoagulant effects of activated protein C, which exists in 2 forms in plasma. Approximately 60 % of PS is bound to the complement component C4b binding protein, and the remaining 40 % is free [65]. PS C4b binding protein has a direct anticoagulant activity, although is less effective than free PS [66]. Recent studies have, in fact, found that free PS is a better indicator of thrombotic events than total PS [67, 68].

It is likely that, compared with other vitamin K-dependent proteins, the blood concentration of PS in this patient was relatively decreased. Although PS is a vitamin K-dependent coagulation factor, the antigen and activity of which are significantly reduced upon the initiation of warfarin therapy [69–71], its concentration is also decreased during long-term anticoagulation with warfarin [72–75]. Consequently, an accurate diagnosis of PS deficiency based on its blood concentrations may be challenging. We recommend comparing the relative changes in PS concentration with that of other vitamin K-dependent proteins, such as prothrombin, factors VII, IX and X. Table 1 lists the plasma factors that influenced this patient’s thrombophilic tendency. It is noteworthy that all measurements of PS concentrations were decreased, whereas the activities of factor X and protein C both remained within normal limits.

**Table 1:** Plasma factors influencing the thrombophilic propensity

Test	Values (percent)	Normal range (percent)
Total protein S antigen	56	65–135
Free protein S antigen	50	60–150
Protein S activity	35	60–150
Protein C activity	89	64–146
Anti-thrombin III activity	95	80–130
Factor X	81	70–130

PS deficiency is a risk factor for deep venous thrombosis and pulmonary thromboembolism, as well as arterial thrombosis, including myocardial infarction [64, 76–79] and, in some studies has been associated with a prominently increased risk of ST [80, 81]. Since the PS activity of patients with a hereditary deficiency is 25 to 40 % of that measured in normal individuals [8] while the PS concentration in patients treated with warfarin is decreased by approximately 50 % [82], it is likely that, in this patient, PS had decreased enough to cause a thrombotic event. The hypercoagulable state due to the low PS concentration measured in this patient seems to have outweighed the anticoagulant effect of warfarin. Thus, she might have developed a significant protein S deficiency and recurrent ST while receiving inadequate doses of warfarin. The discontinuation of warfarin and its replacement by a new antithrombotic agent gradually normalized the PS concentrations, from 64 % at 1 month, to 78 % at 4 months after her discharge from the hospital.

This patient suffered from an accelerated blood coagulation caused by a combination of a) persistent local inflammation due to stent allergy, b) HIT-associated hypercoagulability, and c) warfarin-induced protein S deficiency. The intra-procedural ST was probably caused mainly by HIT after the injection of heparin during PCI. Heparin was replaced by argatroban and warfarin by a new antithrombotic agent. However, ST recurred twice despite the administration of the new antithrombotic and anti-allergic pharmaceutical, though not after the initiation of steroid therapy, suggesting that 1) the thrombotic events were caused mainly by a stent allergy, and 2) PS deficiency and HIT were associated with ST.

## Conclusions and clinical implications

While venous thrombosis and gangrene caused by HIT and PS deficiency have been reported [83, 84], this is the first case of ST associated with metal allergy in addition to HIT and PS deficiency. Our observations suggest that an allergy to the stent should be considered as a possible cause of recurrent ST after stent implantation in relatively young patients, who should undergo patch testing. In addition, a history of metal allergy should be meticulously explored before elective PCI. Besides antiplatelet therapy, steroids may effectively prevent ST caused by a metal allergy. Detailed examinations and the early identification of other aetiologies can prevent recurrent major thrombotic events. It is noteworthy that, once formed, circulating HIT antibodies may be activated by the regular use of heparin-coated vascular access, persist for years, and be the cause of life-threatening thrombotic events. When acute coronary thrombosis occurs during PCI, HIT should always be strongly considered, since the failure to promptly discontinue all heparin administration and initiate an alternate antithrombotic agent may the source of major morbidity and mortality.

### Consent

Written informed consent was obtained from the patient for publication of this Case report and any accompanying images.

## Abbreviations

ST: Stent thrombosis
PS: Protein S
HIT: Heparin-induced thrombocytopenia
ACS: Acute coronary syndrome
pLAD: Proximal left anterior descending
PCI: Percutaneous coronary intervention
ISR: In-stent restenosis
PF4: Platelet factor 4
